# The Interplay of Preoperative Sarcopenia, Systemic Inflammation, and Neoadjuvant Therapy in Resectable NSCLC-Identifying the Gap: A Narrative Review of Surgical and Oncological Outcomes

**DOI:** 10.3390/medicina62050850

**Published:** 2026-04-29

**Authors:** Evangelos Katsiotis, Sofoklis Mitsos, Konstantinos Katsas, Konstantinos Kostopanagiotou, Panagiota Misokalou, Sophia Stamatopoulou, Arezina N. Kasti, Periklis Tomos

**Affiliations:** 1 Department of Nutrition and Dietetics, Attikon University Hospital, 12462 Athens, Greece; gmisokalou@gmail.com (P.M.); sophia.stamatopoulou@hotmail.com (S.S.); kastiare@med.uoa.gr (A.N.K.); 2 School of Medicine, National and Kapodistrian University of Athens, 11527 Athens, Greece; 3 Department of Thoracic Surgery, Medical School, National and Kapodistrian University of Athens, Attikon University Hospital, 12462 Athens, Greece; sophocmit@yahoo.gr (S.M.); kostop@hotmail.co.uk (K.K.); periklistomos@hotmail.com (P.T.)

**Keywords:** sarcopenia, non-small cell lung cancer, neoadjuvant chemoimmunotherapy, systemic inflammation, inflammatory indexes, lung resection surgical outcomes, lung resection oncological outcomes

## Abstract

Preoperative sarcopenia has emerged as an important determinant of adverse postoperative and long-term outcomes in patients with resectable non-small cell lung cancer (NSCLC). Its frequent coexistence with systemic inflammation may further worsen survival outcomes. At the same time, neoadjuvant chemotherapy and chemoimmunotherapy have substantially improved pathological response and survival in resectable NSCLC. However, their interaction with host-related factors such as sarcopenia and systemic inflammatory status remains insufficiently characterized. This narrative review aims to synthesize current evidence regarding the interplay between preoperative sarcopenia, systemic inflammation, and neoadjuvant therapy in resectable NSCLC and evaluates their potential combined impact on surgical and oncological outcomes. A narrative synthesis of 20 studies involving patients undergoing lung cancer resection was performed. Sarcopenia was primarily assessed using computed tomography or PET-CT-derived skeletal muscle indices, most commonly the skeletal muscle index, whereas systemic inflammation was evaluated using biochemical inflammatory markers. The available evidence consistently indicates that preoperative sarcopenia is associated with poorer long-term survival, and this adverse effect appears to be amplified in the presence of systemic inflammation. Although neoadjuvant chemoimmunotherapy has improved tumor response and survival outcomes, it may also act as a systemic stressor capable of aggravating muscle loss. Importantly, no study to date has simultaneously evaluated sarcopenia, systemic inflammation, and neoadjuvant therapy within a unified analytical framework. Most available studies focus primarily on sarcopenia, while inflammatory or treatment-related parameters are typically analyzed separately. Overall, while sarcopenia and systemic inflammation are recognized predictors of adverse outcomes in resectable NSCLC, robust evidence integrating them with neoadjuvant therapy is lacking. Clarifying their potential interaction may improve risk stratification and help to optimize perioperative management strategies in the era of neoadjuvant therapy.

## 1. Introduction

### 1.1. Rationale and Background

Non-small cell lung cancer (NSCLC) poses a major global health challenge and remains a leading cause of cancer mortality [1,2]. For early-stage and locally advanced NSCLC, surgical anatomic resection is the cornerstone of curative-intent treatment [3,4,5]. Patient staging and management depend on the eighth TNM classification, underscoring the importance of complete resection [3,5].

Despite progress in surgical techniques and staging, recurrence risk after curative resection remains high, prompting research into more effective systemic strategies to complement or precede surgery [6,7]. Current perioperative management emphasizes that optimizing oncological success requires more than focusing on tumor traits [4]. Effective care integrates patient biological readiness and resilience—host factors—for major surgery, along with systematic evaluation of modern systemic therapies [4].

### 1.2. Sarcopenia in the Thoracic Surgical Patient

Sarcopenia is not just a theoretical concept but a clinically recognized and measurable condition and is frequently observed in patients undergoing surgical resection for lung cancer. Reported prevalence varies substantially across studies, ranging from approximately 9% to 56%, largely depending on the definition and assessment method used [8]. In a large systematic review using CT-based measurements, the mean prevalence was approximately 43% among patients undergoing lung cancer surgery [9]. Sarcopenia is characterized by a progressive loss of skeletal muscle mass accompanied by declines in muscle strength and physical function [8,9].

### 1.3. Systemic Inflammation as a Prognostic Component

Alongside sarcopenia, systemic inflammation constitutes a critical component of the host–tumor interaction. Cancer-associated inflammation is a recognized hallmark of malignancy that drives metabolic dysregulation and immune suppression. In NSCLC, elevated inflammatory burden—commonly quantified by readily available biochemical indices such as the Neutrophil-to-Lymphocyte Ratio (NLR), the Platelet-to-Lymphocyte Ratio (PLR), and C-reactive protein (CRP)—has been consistently associated with adverse long-term overall and disease-free survival. Furthermore, systemic inflammation acts as a catabolic driver, frequently coexisting with and accelerating muscle depletion, thereby creating a physiological environment that may hinder the efficacy of immune-based therapies [10,11].

### 1.4. The Evolving Landscape of Neoadjuvant Therapy

The management of resectable NSCLC has undergone a paradigm shift with the integration of immunotherapy into the neoadjuvant setting. Moving beyond traditional platinum-based chemotherapy, neoadjuvant chemoimmunotherapy (nCIT) has emerged as the new standard of care for eligible patients (stages II-IIIB). Landmark trials, most notably CheckMate 816, have demonstrated that nCIT significantly outperforms chemotherapy alone, delivering unprecedented rates of pathological complete response (pCR) and extending event-free survival [7,12]. These “oncological gains” define the success of the modern era, offering the promise of systemic control of micrometastatic disease prior to resection.

However, this therapeutic intensification introduces a “grey area” regarding the patient’s physical readiness for surgery. While nCIT successfully downstages the tumor, it simultaneously imposes a dual physiological burden on the host: the known catabolic toxicity of chemotherapy combined with the systemic immune activation of checkpoint inhibitors. This cumulative stress raises concerns that the therapy intended to cure the cancer may inadvertently deplete the patient’s physiological reserves—specifically affecting muscle mass and inflammatory status—thereby altering the risk profile for the subsequent surgery.

### 1.5. The Host–Tumor–Therapy Triad: Key Determinants of Preoperative Resilience

Preoperative assessment of NSCLC patients implies a comprehensive evaluation of three potentially interacting domains that define physiological resilience. First, sarcopenia, representing a critical depletion of metabolic reserve as described above [9]. Second, systemic inflammation, mediated by host-tumor interactions, drives catabolism and suppresses anti-tumor immunity [10,11]. Third, neoadjuvant systemic therapy—encompassing both traditional platinum-based chemotherapy and contemporary chemoimmunotherapy (nCIT)—acts as a cumulative physiological stressor. This therapeutic multimodal approach imposes a composite burden, combining the established cytotoxicity of chemotherapy with the systemic immune activation of checkpoint inhibitors [7,12].

The interplay between these domains is biologically grounded. As synthesized by Deng et al., tumor-derived inflammation depletes immunomodulatory myokines, compromising the host’s response to nCIT [13]. Concurrently, pharmacokinetic disparities—where conventional dosing leads to relative overdosing in muscle-depleted patients—further amplify toxicity risks [14]. However, despite the robust oncological improvements demonstrated by nCIT [7,12], the extent to which this tripartite interaction undermines patient resilience remains unexamined.

### 1.6. Objectives and Narrative Review Strategy

The primary objective of this study was initially to evaluate the interplay of the entire Host–Tumor–Therapy Triad. However, given the scarcity of studies simultaneously evaluating all three factors in a single cohort, this study was designed as a narrative review. Consequently, it adopts a narrative synthesis approach to undertake the following:

1. Systematically analyze sarcopenia as the primary available and measurable determinant of host resilience.
2. Evaluate its associations with systemic inflammation and neoadjuvant therapy outcomes where data exists.
3. Define the critical evidence gap preventing the implementation of a unified preoperative risk stratification model.

This narrative framework is essential for integrating diverse evidence streams to optimize both short-term safety outcomes and long-term survival (Overall Survival and Disease-Free Survival).

The relative analytical depth devoted to each triad component reflects the evidence landscape encountered during the screening process, as will be described in Section 2.2 of this manuscript: while studies focusing solely on sarcopenia were abundant, those investigating its combined effect with systemic inflammation were limited, and studies simultaneously evaluating the full tripartite interaction were virtually non-existent, a reality already acknowledged as an inherent limitation of this review. Within the included studies, this pattern was further confirmed by the distribution of available data (see Section 3). All three components nonetheless receive dedicated and independent analysis in Section 3, with systemic inflammation and neoadjuvant therapy examined as autonomous pillars rather than as modifiers of sarcopenia. What is absent is their integrated evaluation within a unified analytical framework and this constitutes precisely the gap this review seeks to define [15].

## 2. Methods

### 2.1. Literature Search and Study Selection

**Figure 1:**
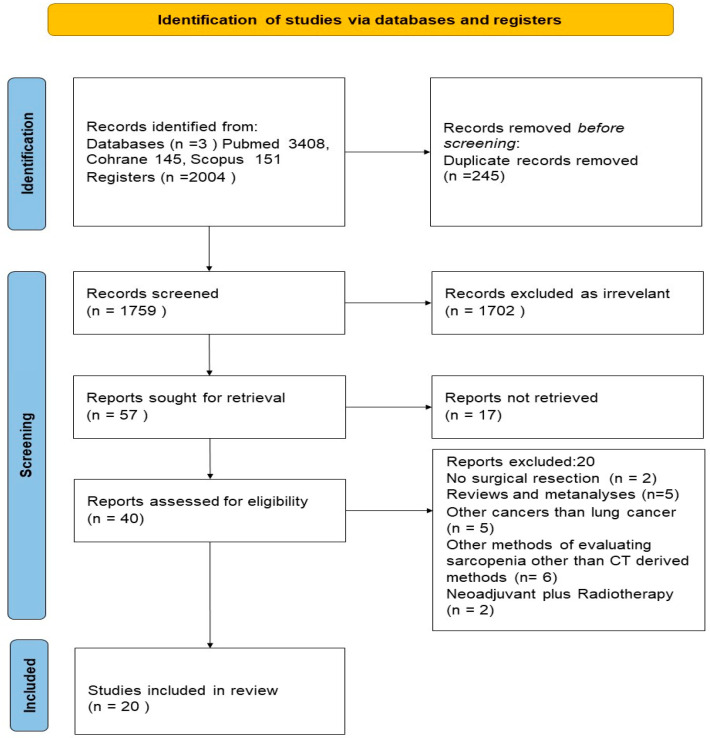
Flow chart: identification and selection of the studies.

This paper is a structured narrative review [16] synthesizing the contemporary literature on the interplay of preoperative sarcopenia, systemic inflammation, and neoadjuvant therapy in resectable NSCLC. A predefined and transparent search process was adopted, and a flow diagram (Figure 1) is provided to illustrate the identification and selection of studies. This does not constitute a systematic review or meta-analysis: no formal risk-of-bias assessment was performed and no quantitative synthesis was conducted. The conclusions represent a narrative interpretation of available evidence and should be read accordingly.

Relevant studies were identified through searches of PubMed, Scopus, and the Cochrane Library. To ensure the retrieval of high-quality evidence, the search was restricted using specific database filters for publication type, including Clinical Trial (Phase I–IV), Comparative Study, Controlled Clinical Trial, Evaluation Study, Multicenter Study, Observational Study, Pragmatic Clinical Trial, Randomized Controlled Trial, and Validation Study. Additionally, filters for English language, Humans, and the publication window (1 January 2021–30 November 2025) were applied. During full-text screening, 17 records were excluded. Although these appeared in search results despite the applied filters, detailed assessment revealed them to be conference abstracts or supplementary materials lacking adequate methodological descriptions for CT analysis (e.g., missing sarcopenia cut-offs). Therefore, they were removed to maintain data quality.

The complete search string, including all Boolean operators and database-specific filters, is provided in full in Table A1 (Appendix A) and is fully reproducible.

Titles and abstracts were screened independently by two authors against the inclusion criteria: (1) original research involving patients undergoing curative-intent resection for NSCLC; (2) assessment of sarcopenia using CT- or PET-CT-based body composition analysis; (3) reporting of overall survival, disease-free survival, or postoperative complications. Discrepancies were resolved through discussion between the two reviewers to reach a consensus.

### 2.2. Adaptation of Review Strategy

The initial objective of this review was to evaluate the preoperative interplay of the entire Host–Tumor–Therapy Triad (sarcopenia, systemic inflammation, and neoadjuvant therapy) and its combined impact on postoperative surgical and oncological outcomes. However, the screening process exposed a significant lack of comprehensive, integrated data: while studies focusing solely on sarcopenia were abundant, those investigating its combined effect with systemic inflammation were limited, and studies simultaneously evaluating the full tripartite interaction were virtually non-existent. Consequently, the review strategy was adapted to reflect this landscape. Sarcopenia was analyzed as the primary, most consistently reported biomarker of host resilience. Where available, its interaction with systemic inflammation and neoadjuvant therapy was synthesized to highlight emerging patterns, ultimately aiming to define the significant knowledge gap preventing the implementation of a unified preoperative risk stratification model.

### 2.3. Exclusion Criteria and Data Quality

Specific exclusion criteria were applied to minimize heterogeneity and ensure data reliability. Neoadjuvant chemoradiotherapy was excluded a priori, as the addition of thoracic radiotherapy introduces distinct patterns of pulmonary toxicity and body composition changes not directly comparable with systemic therapy alone. Similarly, studies were excluded if curative-intent resection was not performed or if cancer histology was other than NSCLC.

To further ensure the reliability of the extracted data—particularly regarding the technical specifications of CT-based body composition analysis (e.g., software, vertebral levels, and cut-offs)—conference abstracts and editorial correspondence were strictly excluded, as detailed in the search strategy. Only full-text original articles published in peer-reviewed journals were retained. Finally, while systematic reviews and meta-analyses were excluded from the formal selection set to avoid data redundancy, they were utilized for background contextualization and manual reference checking. Moreover, their reported outcomes were critically compared with the findings of the present review to further highlight the identified evidence gap.

### 2.4. Final Study Selection

Full-text assessment was sought for 57 records. Of these, 17 were excluded primarily due to the unavailability of a full peer-reviewed manuscript or insufficient methodological detail. Ultimately, 20 original studies met the inclusion criteria and were retained for the final synthesis. Figure 1 illustrates the flow diagram for the identification and selection process.

## 3. Results

### 3.1. Theoretical Framework: The Three Preoperative Pillars of Risk

#### 3.1.1. First Pillar: Sarcopenia

##### The Critical Role of Nutritional Status in Risk Stratification

Nutritional status is now recognized as a crucial factor in determining perioperative risk in cancer surgery [17,18]. Despite its importance, it has historically often been underestimated or neglected within the context of the overall preoperative assessment of patients [17]. Evidence shows that poor nutritional status is associated with worse postoperative outcomes in lung cancer, though findings may vary depending on patient and surgical factors [17].

##### Assessment of Sarcopenia

Sarcopenia is one of the most significant manifestations of malnutrition and overall deterioration of physical health, characterized by the progressive and extensive loss of skeletal muscle mass, accompanied by a decline in muscle strength and functionality [8,9]. This phenomenon is closely associated with the aging process, as well as with chronic pathological conditions, including cancer-related cachexia [8,9]. Accurate and reliable assessment of sarcopenia is a critical element for effective preoperative risk stratification and the appropriate planning of therapeutic interventions [8,9,19]. Computed tomography-based assessment of skeletal muscle mass is widely recognized as a robust method for the evaluation of sarcopenia, with cross-sectional analysis at the level of the third lumbar vertebra (L3) serving as the established reference standard. Measurements obtained at this anatomical landmark demonstrate a strong association with whole-body skeletal muscle mass and enable reliable calculation of the Skeletal Muscle Index (SMI). In oncological cohorts, including patients with lung cancer, low SMI values derived from L3 CT images have been consistently associated with adverse clinical outcomes, underscoring the prognostic relevance of CT-based body composition analysis. As CT imaging is routinely incorporated into cancer staging and follow-up, L3-based muscle assessment provides a feasible and clinically informative approach for early identification of sarcopenia and patient risk stratification [20].

However, considerable heterogeneity exists in how sarcopenia was defined and measured across the included studies, as detailed in Table A2 (Appendix B). The Skeletal Muscle Index calculated from cross-sectional CT images at the third lumbar vertebral level (L3-SMI) was the most frequently employed primary metric, used in five cohorts [20,21,22,23,24]; two further studies incorporated L3 measurements alongside additional thoracic vertebral levels, enabling multi-level comparisons [25,26]. A substantial proportion of cohorts used alternative approaches. Within the lumbar region, the SMI was measured at the first lumbar vertebral level (L1) in one study [27] and at the fourth lumbar vertebral level (L4) in another [28]. Psoas-based constructs were used in three cohorts: psoas muscle area (PMA) assessed by high-resolution CT at L3 [29], and psoas volume index (PVI) in two studies [30,31]. At the thoracic level, three cohorts used cross-sectional muscle area or paraspinous measures at T8–T12 [32,33], and one study used erector spinae muscle area at T12 [34]. Further alternative constructs included paravertebral muscle indices reflecting both quantity and quality [35], intramuscular adipose content alongside SMI without a specified vertebral level [36], a multi-parameter body composition analysis combining SMI with intermuscular and subcutaneous adipose indices [37], a Z-score-based skeletal muscle index integrated with adipose tissue measures [38], and a composite ‘respiratory sarcopenia’ construct incorporating pectoral muscle index and peak expiratory flow rate [39]. Sex-specific threshold values also differed considerably between cohorts, reflecting differences in body composition reference standards across populations [9,19,40]. This methodological diversity—documented in international consensus work on sarcopenia [41]—limits direct comparison of effect sizes and prevalence estimates between studies.

##### The Impact of Sarcopenia on Perioperative and Long-Term Outcomes

The available body of research clearly demonstrates that the presence of sarcopenia is associated with adverse perioperative and long-term outcomes in patients undergoing surgical management of lung cancer [8,19,42,43].

Evidence from systematic reviews and meta-analyses consistently confirms that preoperative sarcopenia is a consistent predictor of adverse outcomes. Specifically, Yang et al. demonstrated that sarcopenia significantly lowers long-term overall survival (HR 2.23, 95% CI: 1.68–2.94) [19]. Regarding surgical safety, a large-scale meta-analysis by Nishimura et al. revealed that sarcopenic patients face a more than two-fold increase in the risk of perioperative complications (e.g., pneumonia, prolonged air leak, atrial fibrillation) with an Odds Ratio of 2.51 (95% CI: 1.55–4.08) [9]. These findings were further corroborated by Kawaguchi et al., who confirmed the association with both postoperative complications (OR 1.86, 95% CI: 1.42–2.44) and poor overall survival (HR 2.89, 95% CI: 2.31–3.62) [8].

The adverse effects of sarcopenia are even more pronounced in patients undergoing combined multimodal therapeutic approaches. In oncological patients receiving combined radiotherapy and/or chemotherapy regimens, the presence of sarcopenia has been associated with a significantly increased risk of adverse events (specifically grade 3 or higher, toxicities, such as myelosuppression, radiation pneumonitis, and esophagitis), with an estimated relative risk (RR) of approximately 1.44 (95% CI: 1.15–1.80) [44]. Simultaneously, sarcopenia has been shown to elevate the overall risk of mortality, as indicated by a hazard ratio (HR) of 1.66 (95% CI: 1.35–2.03) [44]. These data suggest that muscle mass loss may play an active pathophysiological role affecting treatment tolerance and survival [14,44].

#### 3.1.2. Second Pillar: Systemic Inflammation

##### Systemic Inflammation in NSCLC

The interaction between cancer and the host’s immune response is considered fundamental to disease progression [45,46]. The systemic inflammatory response (SIR) plays a central role, as it significantly influences processes such as tumor growth, local invasion, and distant metastases [45,46]. Accordingly, the baseline inflammatory status of a patient constitutes a strong prognostic indicator that significantly influences outcomes in patients with non-small cell lung cancer (NSCLC), particularly those scheduled for surgical treatment [46]. Commonly measured systemic inflammation markers in NSCLC, as well as their prognostic value, are summarized in Table 1.

##### Blood-Based Inflammatory Markers and Their Prognostic Value in Resected NSCLC

CRP, a classic acute-phase reactant, provides a straightforward and immediate assessment of overall systemic inflammatory burden. High preoperative CRP levels, specifically those exceeding a cutoff of >40 mg/L, have been identified as a critical threshold. Lopez-Pastorini et al. demonstrated that levels exceeding >40 mg/L serve as an independent and significant indicator for both elevated postoperative morbidity (*p* < 0.001) and higher mortality (*p* = 0.021) following anatomical lung resection [47].

As summarized in Table 1, NLR and PLR are simple indices calculated from standard complete blood counts and provide an accessible window into systemic inflammatory activity. NLR reflects the balance between neutrophil-driven innate immune responses and lymphocyte-dependent adaptive immunity, while PLR reflects platelet-associated inflammatory—and potentially thrombotic—processes in relation to lymphocyte counts [48,49,50,51].

Reflecting the systemic inflammatory burden, NLR and PLR serve as surrogate markers of the balance between innate/adaptive immunity and platelet-associated inflammation [48,51]. In the specific context of resectable NSCLC, elevated levels of these indices have been consistently validated as independent predictors of adverse oncological outcomes, including reduced recurrence-free and overall survival [46,52].

##### Systemic Immune–Inflammation Index (SII)

The Systemic Immune–Inflammation Index (SII) is a composite score derived from platelet, neutrophil, and lymphocyte counts that reflects systemic inflammatory and immune status. In patients undergoing lung cancer resection, high preoperative SII is independently associated with an increased risk of postoperative pulmonary complications (PPCs) (OR 2.77, 95% CI: 1.08–7.16), alongside established clinical factors such as reduced forced expiratory volume in 1 s (FEV1) [53]. Furthermore, high preoperative inflammation-based scores, including SII and the C-reactive protein-to-albumin ratio (CAR), have been associated with an increased risk of early tumor recurrence following lung cancer resection. Specifically, elevated CAR has been identified as an independent predictor of recurrence-free survival (HR 1.99, 95% CI: 1.20–3.28) [54]. Collectively, SII serves as a composite surrogate marker reflecting the balance between cancer-associated inflammatory activity and host immune status, integrating platelet, neutrophil, and lymphocyte components [53,54].

##### Prognostic Implications of NLR, PLR, and SII

In NSCLC, inflammatory cell-derived markers such as NLR, PLR, and SII have gained attention as indicators of host–tumor interactions. High values typically reflect a pattern of increased circulating neutrophils and platelets together with relative lymphopenia. This profile is associated with a biological environment that favors tumor progression and impaired anti-tumor immune surveillance, as outlined in reviews of biological mechanisms with clinical relevance integrating preclinical evidence [10,11].

Clinical studies in patients undergoing lung cancer resection reinforce the prognostic relevance of these inflammatory indices. Observational data show that higher preoperative NLR, PLR, and SII are associated with poorer recurrence-free and overall survival following surgery. Specifically, Mazzella et al. identified elevated preoperative levels of these markers as significant predictors of mortality, reporting Hazard Ratios of 2.14 (95% CI: 1.17–3.93) for NLR, 2.27 (95% CI: 1.28–4.02) for PLR, and 2.59 (95% CI: 1.53–4.38) for SII [46]. Similarly, in a large cohort analysis, H.-L. Wu et al., validated preoperative NLR as an independent predictor for both recurrence-free (HR 1.27, 95% CI: 1.06–1.51) and overall survival (HR 1.36, 95% CI: 1.07–1.72) [52]. Beyond long-term survival, these markers also impact short-term recovery; specifically, elevated SII has been identified as a strong independent predictor of postoperative pulmonary complications (OR 2.77, 95% CI: 1.08–7.16) [53].

In addition, certain inflammatory markers—particularly SII—have been linked to an increased likelihood of postoperative pulmonary complications after lung resection, highlighting their potential utility in perioperative risk stratification [53]. These findings underscore that systemic inflammation, as reflected by blood-based indices, is not only a feature of tumor biology but also a predictor of postoperative vulnerability.

Overall, evidence from autoimmune, infectious, and oncologic research demonstrates that NLR and PLR are informative, low-cost markers of inflammatory status. In NSCLC, elevated NLR, PLR, and SII are associated with less favorable oncologic outcomes and, in some cases, with higher risk of postoperative pulmonary complications.

##### Prognostic Value of Prognostic Nutritional Index (PNI) in Resected NSCLC

To objectively measure the systemic, combined impact of inflammation and nutritional status, composite indices have been established. PNI is calculated based on two readily available parameters: serum albumin levels and the total peripheral blood lymphocyte count [55,56]. Multiple studies have consistently associated a low preoperative PNI value (commonly defined as <50) with an increased incidence of postoperative complications—particularly infectious morbidities—and significantly reduced overall and recurrence-free survival in patients who have undergone complete resection of NSCLC [55,56,57]. For instance, one study specifically reported an HR of 2.18 (95% CI: 1.08–4.21) for overall survival and 2.57 (95% CI: 1.46–4.38) for recurrence-free survival [56]. Similarly, Park et al. demonstrated that low PNI is independently associated with both poorer overall survival (HR 1.5, 95% CI: 1.2–1.8) and an increased risk of postoperative pulmonary complications (OR 1.7, 95% CI: 1.3–2.3) [57].

##### Integration of Sarcopenia and Inflammatory Status: Synergistic Risk

An increasingly important area of research focuses on the prognostic synergy arising from the combination of anatomical nutritional status (i.e., sarcopenia, as assessed via CT imaging) and biochemical immune–nutritional status (i.e., PNI, which reflects systemic inflammation via lymphocyte count and albumin levels) [21]. The combined assessment of skeletal muscle mass and serum inflammatory nutritional markers is particularly valuable, as it simultaneously considers multiple parameters of patient’s physiological reserves [21].

Recent studies confirm that the coexistence of sarcopenia and systemic immuno-nutritional dysfunction exerts a synergistically negative effect on the prognosis of surgical patients [21]. Specifically, the patient group characterized by the combination of “Sarcopenia and Low PNI” exhibited particularly poor outcomes. This group demonstrated significantly higher rates of both severe respiratory (38.5% vs. 15.0%, *p* = 0.006) and overall complications (53.8% vs. 31.4%, *p* = 0.02) compared with patients who did not present these combined risk factors. Furthermore, multivariate analysis identified this combined status as an independent predictor of respiratory complications (OR 3.23, 95% CI: 1.25–8.35) [21]. These findings underscore the critical need for the simultaneous evaluation of multiple factors during preoperative assessment to enable a more accurate quantification of risk [21].

Additional observations support this synergistic model. Chang et al. demonstrated that systemic inflammation, assessed via NLR, may be a stronger prognostic determinant for overall survival than muscle mass alone (HR 2.04, 95% CI: 1.33–3.22 for NLR versus non-significant *p* = 0.113 for sarcopenia) [22]. Conversely, Sato et al. showed that low skeletal muscle index and low PNI independently worsen survival, reinforcing their biological complementarity (HR 1.85, 95% CI: 1.09–3.14 for low SMI and HR 2.03, 95% CI: 1.23–3.35 for low PNI) [27].

Collectively, these data highlight the need for multidimensional preoperative risk stratification that integrates structural, biochemical, and immunological parameters. Such an approach may enable more accurate identification of high-risk patients and support the development of targeted prehabilitation strategies aimed at modulating inflammation, preserving muscle mass, and improving overall resilience before curative-intent lung cancer surgery.

##### Integration of Inflammatory Markers with Sarcopenia and Neoadjuvant Therapy Context

The prognostic findings described in this section cannot be fully understood in isolation from the structural data on sarcopenia reviewed in the preceding subsection. Chronic elevation of pro-inflammatory cytokines, particularly Tumor Necrosis Factor (TNF) and Interleukin 6 (IL-6), may activate muscle proteolytic pathways, contributing to progressive skeletal muscle depletion [58]. Conversely, loss of skeletal muscle disrupts normal myokine secretion; myokines produced by active muscle tissue exert immunomodulatory and anti-inflammatory effects, and progressive loss of skeletal muscle mass may impair these signalling pathways, potentially amplifying the systemic inflammatory response [13]. This bidirectional relationship is supported by clinical data. Sato et al. [27] reported that low SMI was independently associated with worse five-year overall survival (66.0% vs. 82.2%, *p* = 0.004; HR 1.850, *p* = 0.022), with low PNI emerging as a separate independent prognostic factor in the same multivariate model (HR 2.031, *p* = 0.006), suggesting that muscle depletion and systemic immunonutritional impairment contribute independently to adverse outcomes. Uchibori et al. [21] further demonstrated that the composite of preoperative sarcopenia and immunonutritional impairment was an independent prognostic factor on multivariate analysis, with a five-year survival rate of 52.8% in the combined deficit group (*p* < 0.001), underscoring the particular vulnerability of patients in whom both deficits coexist. The neoadjuvant therapy context introduces further complexity: platinum-based chemotherapy is associated with haematological toxicities including neutropenia [59], which directly affects the cellular components from which ratio-based inflammatory indices such as NLR are derived, potentially altering their values when measured after treatment initiation. Immune checkpoint inhibitors carry a distinct spectrum of immune-related adverse events—including pneumonitis, colitis, hepatitis, and endocrinopathies—that require specific monitoring and management [60]. Preoperative inflammatory indices, body composition metrics, and immunonutritional scores should therefore be evaluated as components of a composite host phenotype rather than as independent univariable predictors [21], particularly in patients scheduled for intensive neoadjuvant regimens where the integration of nutritional, inflammatory, and immunological assessment may be most clinically relevant [15].

#### 3.1.3. Third Pillar: Neoadjuvant Chemotherapy and Chemoimmunotherapy

##### Neoadjuvant Systemic Therapy in Resectable NSCLC

In resectable NSCLC patients, systemic therapy—comprising platinum-based chemotherapy or combined chemoimmunotherapy—can be delivered either after surgery (adjuvant) or before surgery as neoadjuvant treatment. Neoadjuvant therapy is administered preoperatively with the intent to reduce primary tumor burden, facilitate nodal downstaging, increase the likelihood of complete (R0) resection, and eradicate micrometastatic disease at an early stage, while also providing an in vivo assessment of treatment response that may correlate with long-term outcomes such as event-free survival (EFS) and overall survival (OS) [61,62].

Neoadjuvant systemic strategies, when combined with surgery, have been shown to improve DFS and OS compared with surgery alone [61,62,63,64]. Accordingly, leading professional societies, including the American College of Chest Physicians and the Society of Thoracic Surgeons, endorse the use of neoadjuvant systemic therapy in resectable NSCLC, with treatment selection guided by tumor stage, molecular profile, comorbidity burden, and patient-specific factors [4,63].

Current US guidelines favor neoadjuvant chemoimmunotherapy (nCIT) for stage II–IIIB NSCLC, particularly in patients with elevated PD-L1 expression, due to higher rates of pathological complete response and the potential for improved long-term survival [12]. Consequently, professional societies emphasize the personalization of these preoperative approaches based on tumor biology and patient status [4,63].

nCIT provides superior oncologic outcomes compared with neoadjuvant chemotherapy alone in patients with resectable NSCLC. In the phase III CheckMate 816 trial, neoadjuvant nivolumab combined with chemotherapy resulted in a 5-year OS rate of 65.4% versus 55.0% for chemotherapy alone, with a significant reduction in the risk of recurrence or death as reflected in event-free survival analyses [7,65].

In addition, nCIT is associated with substantially higher rates of major pathological response (MPR), a recognized surrogate marker for long-term survival, with reported MPR rates reaching up to 49.3% compared with 19.0% in chemotherapy-only regimens [66]. Randomized trials such as CheckMate 816 and KEYNOTE-671 have consistently demonstrated higher rates of pathological complete response (pCR), prolonged EFS, and improved OS with nCIT compared with chemotherapy alone [7,12].

##### Treatment-Related Catabolic and Inflammatory Stress in the Sarcopenic Host

Although nCIT preserves a generally favorable perioperative safety profile, it is associated with a distinct spectrum of treatment-related toxicities. Neoadjuvant chemotherapy is primarily associated with classical cytotoxic adverse events, including neutropenia, anemia, thrombocytopenia, gastrointestinal toxicity, fatigue, alopecia, and peripheral neuropathy, with grade 3–4 adverse events reported in up to 33% of patients depending on regimen and treatment intensity [67]. In contrast, specific nCIT regimens have demonstrated manageable safety profiles, with grade 3–4 toxicity rates ranging from 18% to 22% in some trials [59,68].

The addition of immune checkpoint inhibitors introduces immune-related adverse events such as pneumonitis, colitis, hepatitis, endocrinopathies, and dermatologic reactions, which may delay or preclude planned surgical procedures [68,69]. These immune-mediated toxicities are driven by immune activation, cytokine release, and tissue inflammation and may be accompanied by phenomena such as nodal immune flare and increased fibrosis, potentially complicating surgical dissection, such as extended surgery time, without substantially increasing perioperative complication rates [59,67,68,70,71,72,73]. A comparative analysis of treatment-related adverse events and surgical implications between neoadjuvant chemotherapy and chemoimmunotherapy in resectable NSCLC is summarized in Table 2.

In patients with preexisting sarcopenia or heightened systemic inflammation, these treatment-related insults may exert disproportionate catabolic and inflammatory effects, leading to treatment interruptions, delays in proceeding to surgery, or impaired postoperative recovery. Through these mechanisms, neoadjuvant therapy–induced stress may indirectly influence long-term oncologic outcomes, including OS and DFS, by modifying treatment delivery, surgical fitness, and perioperative resilience in the above patients.

##### Lack of Host-Factor Stratification in Contemporary nCIT Trials

Current high-impact nCIT trials, such as CheckMate 816, focus primarily on pathological response and survival but lack systematic stratification based on baseline sarcopenia or immuno-nutritional or inflammatory status [7,72,74]. CT-derived skeletal muscle indices, inflammatory markers such NLR, PLR, and CRP-related metrics, as well as composite scores like PNI, are rarely included in prespecified subgroup analyses, despite their known prognostic value in thoracic oncology.

While technical difficulty in surgery and postoperative recovery, following nCIT is well-documented [75], the specific influence of sarcopenia on these outcomes remains largely unexplored. Furthermore, a significant and largely unexplored gap persists regarding the synergistic relationship between sarcopenia, systemic inflammation, and the use of neoadjuvant therapy before surgery on critical long-term outcomes in patients with lung cancer [15]. This lack of evidence limits the development of individualized risk management strategies and hampers accurate risk stratification when selecting patients for intensive neoadjuvant therapy.

### 3.2. Key Findings of Reviewed Studies

Figure 1 presents the flow diagram illustrating the process of study identification and selection for the review. A total of 2004 records were identified through electronic databases, including 1708 from PubMed, 145 from the Cochrane Library, and 151 from Scopus. After removal of 245 duplicates, 1759 titles and abstracts were screened, resulting in the exclusion of 1702 records as irrelevant to the research question. Screening and full-text assessment were performed by two reviewers, applying predefined inclusion and exclusion criteria consistently throughout the process. Full texts were sought for 57 articles, of which 17 were excluded as they were conference abstracts or meeting reports without available full-text data necessary for quality assessment. As a result, 40 studies underwent full-text assessment for eligibility. Of these, 20 were excluded for the following reasons: absence of surgical resection (n = 2), review or meta-analysis articles (n = 5), cancer types other than lung cancer (n = 5), use of non-CT-based methods for sarcopenia assessment (n = 6), and combination of neoadjuvant treatment with radiotherapy (n = 2). Ultimately, 20 original studies met the inclusion criteria and were included in the present review.

As shown in Table 3, over the past five years, multiple observational cohorts from Europe, Asia, and North America have consistently identified preoperative sarcopenia as a negative prognostic factor in resectable NSCLC. Although several of these studies also assessed systemic inflammatory markers and/or recorded neoadjuvant therapy, these factors were rarely analyzed in an integrated manner or formally correlated with sarcopenia.

Several cross-study patterns and methodological inconsistencies merit direct comment before the study-level findings are presented. Sarcopenia was assessed using markedly different CT-based constructs across the twenty cohorts, as documented in detail in Table A2 (Appendix B); this diversity limits direct comparison of effect sizes between studies and should be borne in mind when interpreting the hazard ratios reported below. While the direction of the prognostic effect on overall survival was consistent across most cohorts, two studies reported non-significant associations, Chang et al. (HR 1.421, *p* = 0.113) [22] and Hasenauer et al. (HR 1.27, *p* = 0.240) [20], suggesting that the magnitude of this association may vary depending on surgical setting, patient population, and measurement approach. Inflammatory and immunonutritional markers were reported in only 7 of 20 cohorts and were rarely integrated into formal multivariable models alongside sarcopenia, while neoadjuvant therapy data were excluded or not analytically incorporated in the majority of studies, a pattern that reflects the fragmented, domain-specific approach characterizing the current literature. Geographic concentration and its implications for threshold generalizability are addressed in Section 4.6. Finally, adjustment for key confounders—including surgical approach, histological subtype, disease stage, and neoadjuvant therapy exposure—was inconsistent across studies, limiting causal inference [76].

Across studies using thoracic or lumbar CT/PET-CT-derived measures—such as thoracic skeletal muscle area at T5–T12, psoas volume/area, paravertebral indices, or pectoral muscle measures—sarcopenia was consistently associated with adverse clinical outcomes. Specifically, it demonstrated a significant prognostic impact on poorer overall survival, with Hazard Ratios (HRs) ranging from 1.42 to 1.98 in representative cohorts [21,22,28,29,32,35,39].

Furthermore, sarcopenia was associated with reduced disease-free and recurrence-free survival (HR ranging from 1.39 to 1.47) [22,28] and worse cancer-specific survival (HR 1.74, *p* = 0.008) [32]. Regarding surgical outcomes, in two studies, sarcopenic patients exhibited a significantly increased risk of postoperative complications (Hasenauer et al., 53.2% vs. 39.2%, *p* = 0.017 and Takahashi et al., Odds Ratio 21.00, *p* < 0.001), particularly pulmonary complications such as pneumonia and prolonged air leak [20,29].

These findings were reproduced in heterogeneous settings, including elderly cohorts [30], early-stage disease, minimally invasive resections [20], and innovative constructs such as “respiratory sarcopenia,” which combined pectoral muscle measures with peak expiratory flow rate and was likewise associated with higher complication rates and inferior survival [39].

Only a minority of studies reported inflammatory or immunonutritional markers together with sarcopenia, and these factors were rarely analyzed in an integrated or model-based fashion. However, where assessed, indices such as CRP, NLR, PLR, and PNI frequently emerged as independent predictors of adverse outcomes, sometimes exceeding the prognostic strength of muscle metrics.

For instance, Chang et al. demonstrated that an elevated Neutrophil-to-Lymphocyte Ratio (NLR) was a stronger predictor of inferior Overall Survival (HR 2.04, *p* = 0.001) and Disease-Free Survival (HR 1.714, *p* = 0.003) compared to sarcopenia, which showed a weaker or non-significant association in the same cohort (OS: HR 1.421, *p* = 0.113; DFS: HR 1.392, *p* = 0.034) [22]. Uchibori et al. [21] reported that the composite of sarcopenia and immunonutritional impairment was an independent prognostic factor on multivariable analysis (5-year OS: 52.8%, *p* < 0.001). Sato et al. [27] further corroborated this link, showing that patients with compromised immunonutritional status and muscle depletion experienced significantly worse 5-year survival rates compared to those without (66.0% vs. 82.2%, *p* = 0.004).

Nevertheless, despite these quantitative signals, inflammatory markers were often handled descriptively or entered into models without formal exploration of their synergistic interaction with sarcopenia, leaving the mechanistic interplay between muscle depletion and systemic inflammation only partially elucidated.

Handling of systemic therapy was similarly heterogeneous and often limited. Several cohorts excluded patients who received neoadjuvant chemotherapy or chemoimmunotherapy altogether [23,25,30,34], while others recorded neoadjuvant therapy but did not integrate these variables into multivariable analyses [24,33].

Collectively, these studies demonstrate that while sarcopenia is repeatedly identified as a prognostic factor for OS, DFS, and postoperative complications, the failure to integrate inflammation and neoadjuvant therapy parameters prevents a comprehensive understanding of their combined effect. The limited inclusion of inflammatory markers and the inconsistent handling of Neoadjuvant systemic therapy data undermine the development of robust predictive models. No study assessed all three factors simultaneously; this underscores a gap in current research regarding the combined impact of these parameters on long-term postoperative outcomes. Future research should therefore prioritize integrated designs that simultaneously evaluate sarcopenia, inflammation, and preoperative therapy (neoadjuvant) exposure to elucidate their complex interactions and refine prognostic stratification in NSCLC surgery.

## 4. Discussion

### 4.1. Prognostic Significance of Preoperative Sarcopenia in Resectable NSCLC

The present narrative review confirms that preoperative sarcopenia is consistently associated with poorer long-term oncologic outcomes and higher postoperative morbidity in patients undergoing resection for NSCLC. Across 20 observational cohorts from Asia, Europe and North America, using diverse CT- or PET-CT-based measurements of skeletal muscle at thoracic and lumbar levels (including T8–T12, L3 and pectoral indices), low muscle mass repeatedly emerged as a negative prognostic factor for OS, DFS/RFS and postoperative complications [20,21,22,23,24,25,26,27,28,29,30,31,32,33,34,35,36,37,38,39]. These findings span early-stage and stage I–IIIA disease, elderly cohorts, minimally invasive resections and the newer concept of “respiratory sarcopenia” defined by both pectoral muscle index and peak expiratory flow [39], supporting the interpretation of sarcopenia as a generalizable host-vulnerability phenotype rather than a measurement artifact. Sarcopenia constitutes an independent factor that negatively impacts immediate perioperative morbidity and is significantly associated with reduced long-term survival, as documented by studies [9,19,20]. Moreover, sarcopenia is recognized as a particularly reliable prognostic indicator for tumor recurrence following surgical intervention in patients with non-small cell lung cancer [40].

Accurate and reliable assessment of sarcopenia is a critical element for effective preoperative risk stratification and the appropriate planning of therapeutic interventions [8,9,19]. Traditionally, the “gold standard” for diagnosing sarcopenia has relied on imaging via computed tomography (CT) at the level of the third lumbar vertebra (L3), as this region shows a strong correlation with total skeletal muscle mass. However, more recent methodological approaches have highlighted the increasing practicality and clinical value of thoracic CT assessment [20]. This adapted method focuses on the level of the tenth thoracic vertebra (T10), allowing detailed measurements of both SMI, which reflects the quantity of muscle mass, and Skeletal Muscle Radiation Attenuation (SMRA), used as an indicator of muscle quality through the assessment of fatty infiltration [20]. Incorporating these CT-derived measurements enables clinicians to apply more precise diagnostic criteria for sarcopenia and to identify patients at increased risk at an early stage [20].

Nonetheless, most of these studies evaluated sarcopenia largely in isolation. Systemic inflammatory status and neoadjuvant therapy exposure were frequently not collected, explicitly excluded (for example by omitting all patients who received neoadjuvant chemotherapy) or handled only as crude binary covariates [20,23,24,25,26,28,33,34,35,37,38]. This fragmented approach limits causal inference and leaves unresolved whether sarcopenia itself drives risk, or whether it is a surrogate for broader systemic processes, including chronic inflammation, treatment-related toxicity or advanced biological disease.

### 4.2. Systemic Inflammation and the Sarcopenic Host Phenotype

In the smaller subset of studies that incorporated inflammatory or immune–nutritional indices alongside muscle metrics, a more complex host phenotype becomes apparent. Sato et al. [27] demonstrated that low SMI and low PNI were separate independent prognostic factors, while Uchibori et al. [21] showed that the composite of sarcopenia and immunonutritional impairment was an independent predictor of inferior OS. Chang et al. reported that elevated NLR and PLR are strongly associated with worse OS and DFS, whereas sarcopenia alone displayed only a borderline effect [22]. Studies by Kamigaichi et al., Sun et al. and Takashima et al., also suggest that combinations of structural muscle depletion and inflammatory/immune–nutritional derangements identify particularly vulnerable patients [30,36,39].

Elevated NLR, PLR, and SII are associated with less favorable oncologic outcomes and, in some cases, with higher risk of postoperative pulmonary complications. Although proposed mechanistic explanations exist, the associations observed in surgical cohorts remain observational. Even so, these indices provide useful adjuncts to established clinical and pathological factors when assessing prognosis and perioperative risk in lung cancer patients [10,46,52].

Taken together, these data support the concept that sarcopenia and systemic inflammation form an adverse metabolic–immune phenotype that is especially susceptible to surgical stress, postoperative complications and tumor recurrence. However, even in these more advanced analyses, inflammatory markers are often collected inconsistently and modelled heterogeneously, frequently without explicit testing of interaction or mediation between sarcopenia and inflammation [30,36,39]. As a result, the magnitude and direction of the interplay—whether additive, synergistic or mainly mediated by inflammation—remain incompletely defined.

### 4.3. Evidence Gap and the Need for Multivariable Three-Factor Models (Sarcopenia, Inflammation, Neoadjuvant Therapy)

Across both primary studies and existing reviews (discussed in a subsequent paragraph), the most striking commonality is the near-complete absence of integrative multivariable models that simultaneously consider sarcopenia, systemic inflammation and neoadjuvant therapy when predicting OS and DFS in resectable NSCLC. Lung cancer-specific meta-analyses and broader surgical-oncology reviews typically pool hazard ratios for sarcopenia adjusted for stage, age and performance status, but do not include inflammatory markers or detailed neoadjuvant treatment variables in their multivariable frameworks [19,76,77,78,79,80].

From a methodological standpoint, the field has therefore moved decisively from asking whether sarcopenia is prognostic to quantifying how strongly sarcopenia is associated with adverse outcomes. What remains largely unaddressed—and constitutes the primary evidence gap identified by this review—is the clinical interplay of the tripartite relationship between sarcopenia, systemic inflammation, and neoadjuvant therapy. Specifically, the question is whether the established prognostic value of sarcopenia remains robust, is exacerbated, or is mitigated when accounting for the concurrent burden of systemic inflammation and the physiological effects of modern neoadjuvant therapy. Existing two-factor analyses, in which sarcopenia is combined with PNI, NLR or other inflammatory indices [21,22,27], provide an important intermediate step and suggest that a combined muscle–inflammation phenotype is more informative than either parameter alone. However, they still do not incorporate neoadjuvant treatment in a way that allows the construction of three-factor models.

Sarcopenia in patients with resectable NSCLC is mechanistically linked to poor surgical and oncologic outcomes through several pathways. Loss of skeletal muscle mass and strength impairs functional capacity, reduces metabolic and protein reserves, and alters myokine and immune signaling, thereby diminishing the ability to withstand surgical stress and systemic therapy. Systemic inflammation further promotes catabolism, immune dysregulation, and tumor progression, creating a vicious cycle that exacerbates muscle wasting and impairs recovery [58,81].

Neoadjuvant chemotherapy and immunotherapy, while improving pathological response and survival outcomes (EFS and OS), may aggravate muscle wasting, anorexia, fatigue, and inflammatory burden, further compromising outcomes in vulnerable patients. Sarcopenia is associated with increased toxicity and poorer response to systemic therapies in patients with solid tumors, including NSCLC. This comprises reduced tolerance to immunotherapy, likely due to impaired immune function and altered inflammatory signaling [13,14]. Furthermore, a critical pharmacokinetic mismatch likely contributes to this vulnerability. Since chemotherapy dosing is conventionally calculated based on Body Surface Area (BSA) rather than muscle mass, sarcopenic patients—who possess a reduced volume of distribution for hydrophilic drugs—are inadvertently exposed to higher plasma concentrations relative to their lean body mass. This leads to a ‘relative overdose’ that exacerbates systemic toxicity despite standard BSA-based dosing [14]. nCIT related toxicity is exacerbated in sarcopenic and frail patients, who have reduced physiologic reserve and are more susceptible to adverse events, including infections and delayed recovery [21,22]. Neoadjuvant chemotherapy is dominated by cytotoxic toxicities, whereas neoadjuvant chemoimmunotherapy adds immune-mediated inflammation and irAEs to the toxicity profile, with similar overall rates of severe adverse events but a broader mechanistic spectrum [59,67,68,69,70,71,72,73].

Neoadjuvant therapy in NSCLC increases the length of surgery due to treatment-induced inflammatory changes and fibrosis, particularly with neoadjuvant immunotherapy and chemoimmunotherapy. This effect is well-documented, with higher rates of conversion from minimally invasive to open thoracotomy and longer operative times required to manage dense adhesions and altered tissue planes [68]. In sarcopenic patients, the impact of prolonged surgery is clinically significant. Sarcopenia independently predicts increased postoperative complications, longer hospital stays, and reduced overall survival after lung cancer resection [8,21,76,82]. The combination of sarcopenia and neoadjuvant therapy—both of which can impair nutritional and functional status—may further exacerbate vulnerability to perioperative morbidity, delayed recovery, and poor long-term outcomes.

Whether these three factors interact in a clinically meaningful way remains an open empirical question: no study has yet evaluated sarcopenia, systemic inflammation, and neoadjuvant therapy concurrently within a single analytical framework. The considerations below are grounded in component-specific evidence and should be understood as hypothesis-generating rather than as established causal relationships. Reduced skeletal muscle mass has been consistently associated with impaired ability to tolerate major thoracic surgery in meta-analyses of surgical outcomes in NSCLC [8,9]. Sarcopenia has also been associated with increased toxicity and reduced tolerance to systemic therapies, including chemotherapy, in patients with solid tumors [14,44]. Skeletal muscle functions as an endocrine organ secreting myokines with immunomodulatory and anti-inflammatory effects [13], and sarcopenia has been independently associated with inferior outcomes in patients with solid tumors treated with immune checkpoint inhibitors in a meta-analysis [83], though the extent to which myokine depletion specifically alters immunotherapy response versus serving as a general prognostic marker requires further clarification. Systemic inflammation may in turn accelerate muscle catabolism through pro-inflammatory cytokines, particularly TNF and IL-6, creating a self-reinforcing cycle of muscle depletion that further compromises the patient’s physiological reserve [58]. When neoadjuvant chemoimmunotherapy is subsequently introduced, it imposes a cumulative physiological burden on this already compromised host state: platinum-based cytotoxic agents are associated with myelosuppression, fatigue, and gastrointestinal toxicity, as reported in neoadjuvant NSCLC trials [59,67], while immune checkpoint inhibitors carry a distinct spectrum of immune-related adverse events—including pneumonitis, colitis, hepatitis, and endocrinopathies—that require specific monitoring and management [60]. A pharmacokinetic consideration further compounds risk in sarcopenic patients: standard BSA-based chemotherapy dosing fails to account for body composition, resulting in a relative overdose in those with low muscle mass and amplifying systemic toxicity, as lean body mass may be more relevant than BSA for defining chemotherapy dosing [14,84].

Taken individually, each of these mechanisms is supported by evidence; whether they act synergistically in the same patient—and whether their co-occurrence in the context of modern neoadjuvant regimens translates into clinically meaningful worsening of surgical and oncological outcomes—is precisely what the current evidence base cannot yet determine [21], and addressing this gap is a central motivation for the present review [15].

The key state-of-the-art gap highlighted by this review is therefore the lack of robust, validated multivariable models that integrate all three domains—sarcopenia, systemic inflammation and neoadjuvant therapy—into a single framework for predicting OS, DFS and postoperative complications in surgically treated NSCLC. This gap is also recognized conceptually in broader discussions of host-related factors in oncology [14,15], but has not yet been addressed empirically in the context of resectable NSCLC.

### 4.4. Implications in the Era of Neoadjuvant Chemoimmunotherapy

The rapid adoption of neoadjuvant chemoimmunotherapy (nCIT) in resectable NSCLC adds further urgency to this unmet need. Contemporary trials such as CheckMate 816 and others have demonstrated meaningful improvements in pathological response and survival with nCIT regimens [7,72,74]. However, these studies, and the emerging immuno-oncology review literature, rarely stratify patients according to baseline body-composition or immune–nutritional variables, and almost never evaluate sarcopenia and systemic inflammation together as modifiers of nCIT benefit or perioperative risk.

This oversight is critical because nCIT introduces complex challenges for the Host–Tumor–Therapy Triad. Unlike traditional therapies, nCIT imposes a dual physiological stress: the catabolic burden of the cytotoxic component combined with the systemic inflammatory modulation induced by checkpoint inhibitors. While the mechanisms of systemic therapy-induced wasting are well-established [14], recent data indicate that skeletal muscle mass can significantly decline during the course of neoadjuvant treatment [13]. This creates a clinical paradox: while nCIT successfully downstages the tumor, it may concurrently degrade the patient’s physical resilience—specifically through the aggravation of sarcopenia—potentially offsetting the oncological gains by increasing the risk of adverse postoperative outcomes.

### 4.5. Comparison with Existing Systematic Reviews and Meta-Analyses

The findings of the present review align closely with eight key systematic reviews and meta-analyses that have examined the prognostic role of sarcopenia in cancer, and in lung cancer in particular. In mixed lung cancer populations (including both NSCLC and SCLC and both surgical and non-surgical treatments), Buentzel et al. and Yang et al. demonstrated that CT-assessed sarcopenia is associated with significantly worse OS, with pooled hazard ratios of 3.13 (95% CI: 2.06–4.76) in multivariate analysis and 2.23 (95% CI: 1.68–2.94), respectively [19,77]. Similarly, in a comprehensive meta-analysis focusing specifically on surgically resected NSCLC, Deng et al. [78] concluded that sarcopenia is a strong independent predictor of poor outcomes. Specifically, they reported a Hazard Ratio of 2.85 (95% CI: 1.67–4.86) for Overall Survival and a Risk Ratio of 1.59 (95% CI: 1.01–2.52) for Disease-Free Survival in patients with early-stage disease [78].

A thoracic surgery-focused systematic review by Kawaguchi et al. confirmed that preoperative sarcopenia predicts adverse short- and long-term outcomes, reporting an Odds Ratio of 1.86 (95% CI: 1.42–2.44) for postoperative complications, 1.66 (95% CI: 1.00–2.74) for disease-free survival, and 2.89 (95% CI: 2.31–3.62) for overall survival [8]. These mortality findings were further refined by the recent European Society of Surgical Oncology meta-analysis by Lading et al. [76], which reported a pooled Hazard Ratio of 1.99 (95% CI: 1.73–2.28) for overall survival. Notably, Lading et al. identified an even stronger prognostic effect in patients with early-stage (I–II) disease, reporting an HR of 2.33 (95% CI: 1.91–2.83) [76].

Three broader surgical-oncology reviews provide complementary context. Weerink et al., investigating a diverse cohort of surgical oncology patients—predominantly those with colorectal, hepatopancreatobiliary, and upper gastrointestinal malignancies—reported that low skeletal muscle mass is associated with a significantly higher risk of severe postoperative complications (Odds Ratio 1.44, 95% CI: 1.24–1.68) [80]. Su et al., in gastrointestinal oncology patients predominantly managed with major cancer surgery, found that CT-assessed sarcopenia predicts both short-term postoperative morbidity (Risk Ratio 1.19, 95% CI: 1.08–1.30) and long-term oncologic outcomes, specifically poorer overall survival (Hazard Ratio 1.60, 95% CI: 1.37–1.87) [79]. Finally, a comprehensive pan-surgical meta-analysis demonstrated that preoperative sarcopenia is consistently associated with increased peri- and postoperative mortality (Odds Ratio 2.69, 95% CI: 2.31–3.12), higher complication rates (Odds Ratio 1.68, 95% CI: 1.51–1.87), prolonged hospital stay (Mean Difference 1.68 days), and reduced 1, 3 and 5-year survival (Odds Ratios 0.45, 0.44, and 0.55, respectively) across a wide spectrum of surgical procedures [85].

Collectively, these eight reviews converge with the present synthesis in affirming that preoperative sarcopenia is a strong and clinically meaningful marker of adverse outcomes in lung cancer and in surgical oncology more generally. Crucially, however, across all of them sarcopenia is effectively analyzed as a single host factor. None systematically integrates systemic inflammatory markers into their pooled models, and none explicitly examines interactions between sarcopenia and neoadjuvant regimens with respect to OS, DFS or postoperative complications. The existing review literature therefore robustly answers the question of whether preoperative sarcopenia is prognostic, it clearly is, but does not address how its prognostic impact is modified by preoperative systemic inflammation or contemporary neoadjuvant therapy. The present review complements these prior syntheses by explicitly framing sarcopenia, inflammation and neoadjuvant treatment as a triad of interacting determinants rather than isolated variables. Table 4 provides a comparative overview of key systematic reviews and meta-analyses evaluating the prognostic value of sarcopenia in NSCLC and surgical oncology, highlighting the evidence gap addressed by the present review.

### 4.6. Limitations

This review has several key limitations. The absence of a formal risk-of-bias assessment warrants explicit acknowledgment. Because this is a narrative review, no structured quality-appraisal tool was applied, and evidence from methodologically stronger and weaker studies is synthesized without formal weighting. Among the included cohorts, several are single-centre retrospective studies with relatively small sample sizes—for example, Kamigaichi et al. (n = 98) [36], Cinar et al. (n = 180) [35], and Takahashi et al. (n = 315) [29]—raising concerns about selection bias and limited generalizability. A marked geographic concentration is also evident: 11 of the 20 included studies originated from Asian centres, with nine from Japan, one from Taiwan, and one from South Korea [21,22,23,27,29,30,31,33,34,36,39]. Sarcopenia thresholds are partly population-specific [41], and differences in body composition reference standards between Asian and Western populations may limit the direct applicability of reported cut-off values across clinical settings. These limitations do not negate the consistent directional signal identified across studies, but underscore the need for prospective, geographically diverse, multicentre studies with pre-specified sarcopenia definitions [76].

Narrative reviews are inherently subject to design limitations, including the absence of pre-registered protocols and formal quality appraisal of included studies [16]. The absence of quality weighting means that evidence from methodologically stronger and weaker cohorts is synthesized without formal differentiation. These characteristics are acknowledged; the findings of this review should be regarded as directional and hypothesis-generating rather than definitive.

In addition, because our primary focus is the prognostic impact of sarcopenia, studies were included if sarcopenia was assessed either alone or alongside potential synergistic factors (systemic inflammatory indices and/or neoadjuvant systemic therapy). When such factors were not co-reported, only the effect of sarcopenia was considered, representing an inherent limitation. Furthermore, the search was intentionally limited to English-language publications from 2021 to 2025 to target the nCIT era, which may have excluded relevant evidence and restricted generalizability across populations and time periods. Moreover, the exclusion of conference abstracts and grey literature (n = 17)—necessitated by the need for detailed methodological evaluation of body composition and inflammatory parameters—may have introduced a degree of publication bias. While this criterion ensured that only high quality, peer-reviewed data were analyzed, it is possible that newer, preliminary findings presented solely at conferences were not captured. Substantial clinical and methodological heterogeneity also exists among the 20 included studies, particularly regarding inconsistent definitions and thresholds for sarcopenia (e.g., differing CT measurement levels/cut-offs), as documented in detail in Table A2 (Appendix B) and inflammatory markers (PNI, NLR, PLR). The scope was further narrowed by excluding studies assessing muscle health with non-CT modalities (e.g., handgrip strength, DEXA) and those using neoadjuvant chemoradiotherapy (NACR), limiting the comprehensiveness of body composition assessment and applicability to NACR contexts. This exclusion was based on the premise that thoracic radiotherapy introduces distinct patterns of toxicity, inflammatory response, and body composition change that are not directly comparable to systemic therapy alone. Finally, the proposed synergistic interaction between sarcopenia, systemic inflammation, and neoadjuvant therapy remains largely theoretical because no study evaluated all three domains within an integrated framework, highlighting the need for future prospective research.

### 4.7. Future Directions and Implications for Personalized Surgical Oncology

As demonstrated by the evidence reviewed above, preoperative sarcopenia is a well-established adverse prognostic factor in lung cancer, associated with increased postoperative morbidity and an approximately twofold higher mortality risk. Focusing on resectable NSCLC in the contemporary multimodal treatment era, this review highlights the need to move beyond isolated assessments toward prospective studies using standardized CT-based definitions of sarcopenia and integrated prognostic models that concurrently evaluate muscle status, systemic inflammatory and immune–nutritional indices, and neoadjuvant treatment exposure. These factors should be modelled as interacting and potentially time-dependent predictors of oncologic and surgical outcomes, as their proposed synergy remains largely theoretical. Future studies with broader temporal and geographic representation will be essential to validate these findings across diverse populations and treatment settings. Clinically, incorporating standardized sarcopenia and inflammation metrics into preoperative risk stratification may enable individualized surgical decision-making, targeted perioperative optimization, and more precise, patient-centered care in surgically treated NSCLC following neoadjuvant therapy.

Several practical implications follow from the evidence reviewed. The routine incorporation of CT-derived sarcopenia metrics into the preoperative workup carries no additional burden, since PET/CT—performed skull base to mid-thigh per NCCN (National Comprehensive Cancer Network) guidelines—is recommended as part of pretreatment evaluation across all stages of NSCLC (stages IA through IV), and this field of view inherently includes the L3 vertebral level [86]. The inflammatory indices NLR, PLR, SII, CRP, and PNI can be obtained from routine blood work without incurring additional costs, as they are derived from standard laboratory parameters already collected during patient care [87,88]. In patients with resectable or locally advanced NSCLC and signs of systemic inflammation, a more comprehensive preoperative assessment may be warranted. As detailed in the preceding sections, elevated NLR is independently associated with adverse recurrence-free and overall survival in operable NSCLC [52], and may therefore identify high-risk patients who could benefit from more intensive perioperative evaluation. Beyond PET-CT, other diagnostic tools may be relevant in selected cases. For example, echocardiography may help identify cardiac metastases that can be difficult to detect otherwise: lung cancer is the most common primary site of cardiac metastasis, accounting for approximately 34–36% of all secondary cardiac tumors, yet most cardiac metastases remain clinically silent [89,90,91]. When a cardiac mass is identified, specific echocardiographic parameters, including infiltration pattern, pericardial effusion, sessile implantation, and inhomogeneity, can accurately predict malignancy and are independently associated with lower survival, with direct implications for staging and perioperative management [92]. Systematic integration of body composition and inflammatory profiling into structured preoperative pathways may support targeted prehabilitation, as suggested by recent randomized evidence in sarcopenic and high-risk surgical patients [93,94] and more individualized patient selection for intensive neoadjuvant regimens [4].

## 5. Conclusions

The optimal management of resectable NSCLC requires an integrated approach that considers host biology alongside oncologic therapy [4]. This review demonstrates that while sarcopenia and systemic inflammation are independently prognostic, their interaction with each other and with neoadjuvant therapy is a key determinant of outcomes that remains poorly quantified [21,44]. The complementary prognostic value of inflammatory and immune–nutritional indices alongside muscle metrics supports the model of an adverse host phenotype—where sarcopenia and inflammation are interconnected manifestations—rather than isolated risk factors. The introduction of nCIT represents a significant shift in treatment, providing higher rates of pathological response and improved survival compared to conventional chemotherapy [72,74]. However, sarcopenia, characterized by loss of muscle mass and strength, along with systemic inflammation, can hinder the patient’s ability to tolerate major lung resection and systemic therapy. While neoadjuvant regimens are beneficial on a population level, they may worsen muscle wasting, anorexia, fatigue, and treatment-related inflammation in vulnerable individuals.

Despite this clear biological rationale, the full potential of nCIT remains constrained by the lack of standardized data linking the patient’s baseline biological status—particularly sarcopenia and systemic inflammation—with specific surgical outcomes, postoperative complications, and long-term survival [15].

Current evidence almost uniformly evaluates these domains separately: sarcopenia alone, inflammation alone, or systemic therapy in isolation. Robust, validated multivariable models that integrate all three domains—sarcopenia, systemic inflammation and neoadjuvant therapy—into a single framework for predicting overall survival, disease-free survival and postoperative complications in surgically treated NSCLC are essentially lacking.

Future research should therefore move beyond one-dimensional body-composition metrics and adopt prospective, multidimensional designs that use standardized CT-based definitions of sarcopenia, comprehensive inflammatory and immune–nutritional profiling, and detailed characterization of neoadjuvant regimens. These variables should be modelled as interacting, potentially time-dependent determinants of outcome and linked to structured host-optimization strategies such as nutritional support, immunonutrition and prehabilitation [95]. Prioritizing research that integrates these complex nutritional, inflammatory and immunological factors into unified prognostic models and clinical pathways will enable the medical community to develop personalized, maximally effective therapeutic strategies for each patient with resectable NSCLC, ultimately improving both survival and recovery [15,74,95].

## Figures and Tables

**Table 1:** Markers used for measuring Systemic Inflammation status in NSCLC patients.

Marker	Components/Calculations Methods	Clinical Implication	Prognostic Value for Postoperative NSCLC Outcomes
CRP	Serum C-reactive protein concentration (mg/L)	Overall systemic inflammatory burden; acute-phase response	Preoperative CRP higher than 40 mg/L predicts higher postoperative morbidity and mortality after lung cancer resection.
NLR	Neutrophil count/lymphocyte count	Innate versus adaptive immune balance; systemic inflammation	High preoperative NLR predicts more postoperative pulmonary complications and poorer recurrence-free and overall survival in NSCLC.
PLR	Platelet count/lymphocyte count	Platelet-driven inflammation/thrombosis and immune system status	High PLR is associated with unfavorable recurrence-free and overall survival after NSCLC surgery.
SII	Platelets × neutrophils/lymphocytes	Global balance between systemic inflammation and immune response	High SII predicts a higher risk of postoperative pulmonary complications and shorter survival after lung cancer resection.
PNI	Serum albumin concentration and total lymphocyte count	Combined nutritional reserves and immune competence	Low preoperative PNI (<50) predicts more postoperative complications, prolonged air leak and poorer OS/RFS after NSCLC resection.

Abbreviations. C-reactive protein (CRP); neutrophil-to-lymphocyte ratio (NLR); platelet-to-lymphocyte ratio (PLR); systemic immune–inflammation index (SII); prognostic nutritional index (PNI); non-small cell lung cancer (NSCLC); overall survival (OS); recurrence-free survival (RFS); forced expiratory volume in 1 s (FEV1).

**Table 2:** Comparative analysis of treatment-related adverse events and surgical implications between neoadjuvant chemotherapy and chemoimmunotherapy in resectable NSCLC.

Feature	Neoadjuvant Chemotherapy	Neoadjuvant Chemoimmunotherapy (nCIT)
Common Toxicities	Classic cytotoxic: Neutropenia, anemia, nausea, fatigue, alopecia, and neuropathy.	Combined: Cytotoxic effects + Immune-related adverse events (irAEs).
Grade 3–4 TRAEs	Up to 33% (primarily hematological like neutropenia).	18–22% (e.g., neutropenia, diarrhea, and fatigue).
Unique Risks	Cumulative marrow suppression.	irAEs: Pneumonitis, colitis, hepatitis, and endocrinopathies.
Surgical Impact	Standard recovery expectations.	Potential for delays due to irAEs or “immune flare.”

Abbreviations. Neoadjuvant Chemoimmunotherapy (nCIT); Treatment-Related Adverse Events (TRAEs); Immune-related Adverse Events (irAEs).

**Table 3:** Studies included the review.

Study Details	Patient Characteristics	Main Correlation	Follow-Up (Months)	Methods of Measuring Sarcopenia	Neoadjuvant Screening (Chemo/Ncit)	Inflammation Screening	Surgery Type	Main Outcomes
Huang et al. 2025 [37] China/USA/NL N: 2712	Age: (Mean) 61.5 ± 10.9, Ca Stage: I–IV, Hist: ADC 65.9%	Muscle mass on OS and DFS	NA	SMI, IMAI and SAI by CT or PET-CT	NA	NA	NA	HR 0.86 (95% CI: 0.82–0.90) per unit of increasing SMI
Sun et al. 2025 [39] Japan N: 806	Age: (Median) 69 [64–76], Ca Stage: I–IIIA, Hist: NA	Respiratory Sarcopenia on OS and postoperative complications within 30 days	NA	Respiratory sarcopenia: identified by the presence of a low PEFR. Respiratory sarcopenia diagnosis is confirmed by the additional presence of a low PMI by CT or PET-CT	ΝA	CRP in multivariate analysis	Lobectomy and mediastinal lymph node dissection	Sarc on OS (HR 1.83, *p* = 0.01)
Takashima et al. 2025 [30] Japan N: 334	Age: (Median) 78 [75–87], Ca Stage: 0–III, Hist: ADC 76%, SCC 22%	Sarcopenia on OS and postoperative 90-day complication	41.5 (0.4–136.3)	PVI by CT or PET-CT	Excluded those receiving neoadjuvant	NLR, PNI recorded before surgery but not in multivariable analysis	Pneumonectomy/bilobectomy/lobectomy	5-yr OS: 80.5% vs. 66.7% (Sarc), *p* = 0.012
Verkoulen et al. 2025 [38] Netherlands N: 530	Age: (Mean) 67 ± 9.5, Ca Stage: 0–IV, Hist: ADC 94.9%,	Muscle mass on OS	NA	FEV1%, low Z-SM-index—indicative for a low total body skeletal muscle mass. SM, VAT and SAT index by CT or PET-CT	NA	NA	NA	Higher Z-SM-index associated with higher OS-HR on OS 0.87 (95% CI: 0.77–0.99, *p* = 0.032)
Uchibori et al. 2024 [21] Japan N: 300	Age: (Median) 70 [63–75], Ca Stage: I–IIIA, Hist: ADC 78%	Sarcopenia and immune nutritional status by PNI on OS	64 (58–75)	SMI on L3 level, PMI by CT or PET-CT	ΝA	Immune nutritional status by PNI	Lobectomy	The composite of sarcopenia and low PNI was an independent prognostic factor on multivariable analysis (5-year OS: 52.8%, *p* < 0.001)
Chang et al. 2023 [22] Taiwan N: 298	Age: (Median) 65 [57–73], Ca Stage: I–IIIA, Hist: NA	Sarcopenia and inflammation on OS and DFS	NA	SMI on L3 level by CT or PET-CT	ΝA	NLR, PLR	Lobectomy/wedge/segmentectomy	HR, Sarc on OS: 1.421 (*p* = 0.113), HR, Sarc on DFS: 1.392 (*p* = 0.034), HR, NLR on OS: 2.04 (*p* = 0.001), HR, NLR on DFS: 1.714 (*p* = 0.003)
Hasenauer et al. 2023 [20] Switzerland N: 401	Age: (Mean) 67.1 ± 9.3, Ca Stage: I–III, Hist: ADC 72%	Sarcopenia on OS and postoperative 30-day complications	45 (32.1–69)	SMI on L3 level by CT or PET-CT	ΝA	NA	Lobectomy/segmentectomy by VATS	HR Sarc on OS: 1.27 (*p* = 0.240), Sarc presented higher rates of overall postoperative complications (53.2% vs. 39.2%, *p* = 0.017) and pulmonary complications (48.9% vs. 33.7%, *p* = 0.008)
Kaltenhauser et al. 2023 [26] Germany N: 280	Age: (Median) 66.1, Ca Stage: 0–IV, Hist: ADC 47%, SCC 34%	Sarcopenia on OS and Ca specific survival	55.7 (46.8–71.3)	SMI on L3, T5, T8, T10 levels by CT or PET-CT	NA	NA	Pneumonectomy/lobectomy/segmentectomy	Worse OS in Sarc, *p* < 0.001
Sato et al. 2023 [27] Japan N: 386	Age: (Mean) 68.4 ± 9.1, Ca Stage: I–II, Hist: ADC 79%, Non-ADC 21%	Sarcopenia and immune nutritional status by PNI, on OS and postoperative complications	43.1 (1.5–97.8)	SMI was assessed on L1 level by CT	NA	PNI: calculated from serum albumin level and lymphocyte count, acts as a marker for assessing nutritional and inflammatory status	Lobectomy/segmentectomy	5-yr OS: 66% (Sarc) vs. 82.2%, *p* = 0.004
Vedire et al. 2023 [28] USA N: 492	Age: (Median) 68.5 [61–75], Ca Stage: I–III, Hist: NA	Sarcopenia on OS and RFS	NA	SMI on L4 level by CT or PET-CT	ΝA	NA	Lobectomy	OS (HR 1.65, *p* = 0.001)- RFS (HR = 1.47, *p* = 0.03)
Yamada et al. 2023 [31] Japan N: 645	Age: Sarc 71; Non-sarc 68, Ca Stage: 0–II, Hist: ADC 83%	Sarcopenia on OS and RFS	61 (18–102)	PVI by CT or PET-CT	NA	NA	Lobectomy/sublobar	5-yr OS: 72.4% vs. 88.8%, *p* < 0.001
Cinar et al. 2022 [35] Turkey N: 180	Age: (Median) 65 [36–83], Ca Stage: I–IV, Hist: ADC 53–67%	Sarcopenia on OS	26.3 (1–84)	PVMI and PVMD by CT or PET-CT	ΝA	NA	Lobectomy/sublobar	5-yr OS: PVMI, HR 1.77, *p* = 0.014
Kamigaichi et al. 2022 [36] Japan N: 98	Age: Normal IMAC 67.4; High IMAC 72.1, Ca Stage: I–II, Hist: ADC 83.7%	Sarcopenia on OS	59	IMAC and SMI by CT or PET-CT	NA	NLR PNI (but not included in multivariable analysis)	Lobectomy/segmentectomy	5-yr OS: 82.6% (Sarc) vs. 97.3%, *p* = 0.022 (SMI measuring)
								5-yr OS: 82.4% (Sarc) vs. 97.3%, *p* < 0.001 (IMAC) (95% CI, 90.0–99.3) in patients with normal IMAC 82.4% (95% CI, 61.3–93.2) in patients with high IMAC (*p* < 0.001)
Lee et al. 2022 [23] Korea N: 636	Age: (Median) 61 [54–68], Ca Stage: I–IV, Hist: ADC	Sarcopenia on OS	NA	SMI on L3 level by CT or PET-CT	Excluded those receiving neoadjuvant	NA	Pneumonectomy/lobectomy/wedge	Mean OS: 93.3 (Sarc) vs. 109.4 mo, *p* < 0.001
Ueda et al. 2022 [34] Japan N: 534	Age: Sarc 69.5; Non-sarc 68, Ca Stage: I, Hist: ADC 80%	Sarcopenia on OS	61.5	ESM on T12 level by CT or PET-CT	Excluded those receiving neoadjuvant	NA	Lobectomy/segmentectomy	5-yr OS: 79.6% (Sarc) vs. 89.5%, *p* < 0.001
Wakefield et al. 2022 [25] USA N: 221	Age: (Median) 68.8, Ca Stage: I–II, Hist: ADC	Sarcopenia on OS and DFS and postoperative complications	46.9	SMI: L3, T5, T12 levels by CT or PET-CT	Excluded those receiving neoadjuvant	NA	Anatomical resection	HR on OS varies by level of measuring (T5, T12, L3)
Daffré et al. 2021 [24] France N: 238	Age: (Mean) 63 ± 10.3, Ca Stage: 0–IV, Hist: ADC 31.9%, Non-ADC 68.1%	Sarcopenia on OS	≥60	SMI on L3 level by CT or PET-CT	Neoadjuvant recorded but not correlated in multivariate analysis	NA	Pneumonectomy	5-yr OS: 31.6% (Sarc) vs. 42.6%, RR 1.54
Y. Takahashi et al. 2021 [29] Japan N: 315	Age: (Median) 70 [35–88], Cancer Stage: I, Hist: ADC 73%, Non-ADC 27%	Sarcopenia on OS and postoperative complications	58.8 (0.7–137)	PMA on the L3 level on the HRCT	NA	NA	Lobectomy	5-yr OS: Sarc (vs. non-sarc) HR: 1.978, *p* = 0.01
								Post Operation complications (Sarc): Odds Ratio 21.00, *p* < 0.001
Tanaka et al. 2021 [33] Japan N: 587	Age: (Mean) 68.5 ± 8.8, Ca Stage: 0–III, Hist: ADC 67%, SCC 28.4%	Sarcopenia on OS and DFS and postoperative outcomes	37.2	Paraspinous muscle sarcopenia at the T12 level by CT or PET-CT	Neoadjuvant recorded but not correlated in multivariate analysis	CRP but not in multivariate analysis	Pneumonectomy/lobectomy	HR 1.09 per lower SMI (high SMI means high muscle mass)
Troschel et al. 2021 [32] USA/Germany N: 367	Age: (Median) 62.2 [56–69], Ca Stage: I–IV, Hist: SCC 58%, ADC 33%	Sarcopenia on OS	20.5	T8, T10, T12 muscle CSA by CT or PET-CT	NA	NA	Pneumonectomy	5-yr OS (Ca specific), HR 1.74, *p* = 0.008

Abbreviations. Subcutaneous Adipose Tissue (SAT); Non-Small Cell Lung Cancer (NSCLC); Computed Tomography (CT); Visceral Adipose Tissue (VAT); high-resolution computed tomography (HRCT); Peak Expiratory Flow Rate (PEFR); Pectoral Muscle Index (PMI); Skeletal Muscle Index (SMI); Lumbar vertebrae 1, 3, 4 (L1, L3, L4); Cross-Sectional Area (CSA); Psoas Volume Index (PVI); Positron Emission Tomography/Computed Tomography (PET/CT); Skeletal Muscle tissue (SM); Thoracic vertebrae 8, 5, 12, 10 (T5, T8, T10, T12); Intermuscular Adipose Index (IMAI); Subcutaneous Adipose Index (SAI); Psoas Muscle Index (PMI); Erector Spinae Muscle (ESM); Forced Expiratory Volume in 1 s (percentage) FEV1%; Intramuscular Adipose tissue Content (IMAC); Paravertebral Muscle Index (PVMI); Paravertebral Muscle Density (PVMD); Psoas Muscle Area (PMA); Prognostic Nutritional Index (PNI); Neutrophil-to-Lymphocyte Ratio (NLR); Platelet-to-lymphocyte ratio (PLR); C-Reactive Protein (CRP); Overall Survival (OS); Recurrence-Free Survival (RFS); Disease-Free Survival (DFS); Video-Assisted Thoracoscopic Surgery (VATS); Adenocarcinoma (ADC); Complete tumor removal (R0); Squamous Cell Carcinoma (SCC); Non-Small Cell Lung Cancer (NSCLC); Hazard Ratio (HR); Z-score of SM index (Z-SM-Index); Not Available (NA); Sarcopenic Group (Sarc); Non-sarcopenic group (Non-sarc); Netherlands (NL); Histological Type (Hist); Cancer (Ca); Year (yr); Months (mo); Versus (vs.); Chemotherapy (chemo); Neoadjuvant Chemoimmunotherapy (ncit).

**Table 4:** Comparative overview of key systematic reviews and meta-analyses evaluating the prognostic value of sarcopenia in NSCLC and surgical oncology, highlighting the evidence gap addressed by the present review.

Author (Year)	Target Population	Primary Findings (Sarcopenia Impact)	Integrated with Systemic Inflammation?	Integrated with Neoadjuvant Therapy?
Buentzel et al. (2019) [77]	Mixed Lung Cancer (NSCLC & SCLC)	OS: HR 1.96 (Univariate)/HR 3.13 (Multivariate)	No	No
Yang et al. (2019) [19]	Mixed Lung Cancer	OS: HR 2.23 (95% CI: 1.68–2.94)	No	No
Deng et al. (2019) [78]	Resected NSCLC	OS: HR 2.85 (95% CI: 1.67–4.86) DFS: RR 1.59 (Early-stage only)	No	No
Kawaguchi et al. (2021) [8]	Thoracic Surgery (NSCLC focus)	OS: HR 2.89 (95% CI: 2.31–3.62) Complications: OR 1.86 (95% CI: 1.42–2.44)	No	No
Lading et al. (2025) [76]	Surgically Treated NSCLC	OS: HR 1.99 (95% CI: 1.73–2.28) (HR 2.33 in Stage I–II)	No	No
Weerink et al. (2020) [80]	Surgical Oncology (Mixed)	Severe Complications: OR 1.44 (95% CI: 1.24–1.68) 30-day Mortality: OR 2.15	No	No
Su et al. (2019) [79]	GI & Cancer Surgery	Morbidity: RR 1.19 (95% CI: 1.08–1.30) OS: HR 1.60 (95% CI: 1.37–1.87)	No	No
Knoedler et al. (2023) [85]	Pan-Surgical (General)	Mortality: OR 2.69 (95% CI: 2.31–3.12) Complications: OR 1.68 (95% CI: 1.51–1.87)	No	No
Present Review (2026)	Resectable NSCLC	Identifies the lack of data on the interaction of sarcopenia, neoadjuvant and inflammation.	YES (Analyzed as a key synergistic factor)	YES (Identified as the critical missing variable)

Abbreviations. Non-Small Cell Lung Cancer (NSCLC); Small Cell Lung Cancer (SCLC); Overall Survival (OS); Disease-Free Survival (DFS); Hazard Ratio (HR); Relative Risk (RR); Odds Ratio (OR); 95% Confidence Interval (95% CI); Univariate Analysis (Univariate); Multivariate Analysis (Multivariate); Gastrointestinal (GI).

## Data Availability

No new data were created or analyzed in this study. Data sharing is not applicable to this article.

## Abbreviations

The following abbreviations are used in this manuscript:

ADC	Adenocarcinoma
AE	Adverse Event
ALK	Anaplastic Lymphoma Kinase
BMI	Body Mass Index
BSA	Body Surface Area
CAR	C-reactive Protein-to-Albumin Ratio
CI	Confidence Interval
CRP	C-Reactive Protein
CSA	Cross-Sectional Area
CT	Computed Tomography
DFS	Disease-Free Survival
EFS	Event-Free Survival
EGFR	Epidermal Growth Factor Receptor
ESM	Erector Spinae Muscle
FEV1%	Forced Expiratory Volume in 1 s (percentage)
HR	Hazard Ratio
HRCT	High-Resolution Computed Tomography
IL-6	Interleukin 6
IMAC	Intramuscular Adipose Content
IMAI	Intermuscular Adipose Index
irAEs	Immune-related Adverse Events
L3/T5/T8/T10/T12	Vertebral levels used for muscle measurement (Lumbar/Thoracic)
MPR	Major Pathological Response
nCIT	Neoadjuvant chemoimmunotherapy
NLR	Neutrophil-to-Lymphocyte Ratio
NSCLC	Non-Small Cell Lung Cancer
OR	Odds Ratio
OS	Overall Survival
pCR	Pathological Complete Response
PD-1	Programmed Death-1
PD-L1	Programmed Death-Ligand 1
PEFR	Peak Expiratory Flow Rate
PET	Positron Emission Tomography
PET-CT	Positron Emission Tomography–Computed Tomography
PLR	Platelet-to-Lymphocyte Ratio
PMI	Pectoral Muscle Index
PMA	Psoas Muscle Area
PNI	Prognostic Nutritional Index
PPCs	Postoperative Pulmonary Complications
PRISMA	Preferred Reporting Items for Systematic Reviews and Meta-Analyses
PVMI/PVMD	Paravertebral Muscle Index/Paravertebral Muscle Density
R0	Complete (microscopically margin-negative) tumor resection
RFS	Recurrence-Free Survival
RR	Relative Risk
SCC	Squamous Cell Carcinoma
SII	Systemic Immune–Inflammation Index
SIR	Systemic Inflammatory Response
SMI	Skeletal Muscle Index
SMRA/SMMA/SMA	Skeletal Muscle Radiation Attenuation/Mass Area (as used across studies)
TNF	Tumor Necrosis Factor
TRAEs	Treatment-Related Adverse Events
VATS	Video-Assisted Thoracoscopic Surgery

## Appendix A

**Table A1:** Literature search syntax.

• The ‘date’ filter was utilized for this search (1 January 2021–30 November 2025) • The ‘Human’ filter was utilized for this search • Publication Type Filters Restricted to: ‘Clinical Trial (Phase I–IV), Comparative Study, Controlled Clinical Trial, Evaluation Study, Multicenter Study, Observational Study, Pragmatic Clinical Trial, Randomized Controlled Trial, Validation Study.’ • For the review of research papers: (“Lung cancer” OR “Non-small cell lung cancer” OR “Non-small cell lung cancer” OR “NSCLC” OR “Lung carcinoma” OR “Lung neoplasm” OR “Pulmonary neoplasm” OR “Pulmonary carcinoma” OR “Pulmonary cancer”) AND (“sarcopenia” OR “Muscle wasting” OR “Skeletal muscle index” OR “Skeletal muscle density” OR “Skeletal muscle depletion” OR “Muscle mass” OR “Muscle strength” OR “Muscle atrophy” OR “Muscular atrophy” OR “nutritional status” OR ‘’neoadjuvant chemotherapy’’ OR ‘’neoadjuvant chemoimmunotherapy’’ OR “induction therapy” OR “inflammatory index” OR “NLR” OR “PLR” OR “PNI” OR “SII” OR “CRP” OR “systemic inflammation” OR “surgery” OR “post-operation” OR “perioperative outcomes” OR “lung resection”) NOT (Meta-Analysis OR metaanalysis OR review)

## Appendix B

**Table A2:** CT-based sarcopenia assessment methods and definitions across the included studies.

Study	CT Metric	Anatomical Level	Notes
Huang et al. 2025 [37]	SMI, IMAI, SAI	CT/PET-CT	Multi-parameter body composition analysis; no single vertebral level specified
Sun et al. 2025 [39]	PMI + PEFR	CT/PET-CT	‘Respiratory sarcopenia’ composite construct: low PMI confirmed by low PEFR
Takashima et al. 2025 [30]	PVI	CT/PET-CT	Psoas volume index; elderly cohort (median age 78); neoadjuvant excluded
Verkoulen et al. 2025 [38]	Z-SM-index, SM, VAT, SAT	CT/PET-CT	Z-score-based skeletal muscle index; integrated with FEV1% for pulmonary function
Uchibori et al. 2024 [21]	SMI	L3	Combined assessment with PNI; sarcopenia–immunonutrition interaction model
Chang et al. 2023 [22]	SMI	L3	Combined with NLR and PLR; comparative analysis of muscle vs. inflammation prognosis
Hasenauer et al. 2023 [20]	SMI	L3	Muscle quantity (SMI) and quality (SMRA) assessed; VATS cohort
Kaltenhauser et al. 2023 [26]	SMI	L3, T5, T8, T10	Multi-level comparison; thoracic vs. lumbar SMI at four vertebral levels
Sato et al. 2023 [27]	SMI	L1	L1 level; combined with PNI; early-stage disease (I–II)
Vedire et al. 2023 [28]	SMI	L4	L4 level; no inflammation data reported
Yamada et al. 2023 [31]	PVI	CT/PET-CT	Psoas volume index; early-stage NSCLC (0–II)
Cinar et al. 2022 [35]	PVMI, PVMD	CT/PET-CT	Paravertebral muscle quantity (PVMI) and density (PVMD); muscle quality component
Kamigaichi et al. 2022 [36]	IMAC, SMI	CT/PET-CT	Intramuscular adipose content (IMAC) as a muscle quality metric; stages I–II
Lee et al. 2022 [23]	SMI	L3	Neoadjuvant therapy excluded
Ueda et al. 2022 [34]	ESM area	T12	Erector spinae muscle; stage I only; neoadjuvant excluded
Wakefield et al. 2022 [25]	SMI	L3, T5, T12	Multi-level analysis; neoadjuvant excluded
Daffré et al. 2021 [24]	SMI	L3	Neoadjuvant recorded but not in multivariable analysis
Takahashi et al. 2021 [29]	PMA	L3	Psoas Muscle Area via high-resolution CT; stage I lobectomy cohort
Tanaka et al. 2021 [33]	Paraspinous muscle sarcopenia	T12	Paraspinous/erector spinae construct; neoadjuvant recorded but not correlated
Troschel et al. 2021 [32]	Muscle CSA	T8, T10, T12	Multi-level thoracic CSA; USA/Germany multicentre

Abbreviations. SMI: Skeletal Muscle Index; SMRA: Skeletal Muscle Radiation Attenuation; IMAI: Intermuscular Adipose Index; SAI: Subcutaneous Adipose Index; VAT: Visceral Adipose Tissue; SAT: Subcutaneous Adipose Tissue; Z-SM-index: Z-score of Skeletal Muscle Index; PMI: Pectoral Muscle Index; PEFR: Peak Expiratory Flow Rate; PVI: Psoas Volume Index; PMA: Psoas Muscle Area; ESM: Erector Spinae Muscle; PVMI: Paravertebral Muscle Index; PVMD: Paravertebral Muscle Density; IMAC: Intramuscular Adipose Content; CSA: Cross-Sectional Area; HRCT: High-Resolution Computed Tomography; CT: Computed Tomography; PET-CT: Positron Emission Tomography–Computed Tomography; L1/L3/L4: Lumbar vertebrae 1, 3, 4; T5/T8/T10/T12: Thoracic vertebrae 5, 8, 10, 12; PNI: Prognostic Nutritional Index; NLR: Neutrophil-to-Lymphocyte Ratio; PLR: Platelet-to-Lymphocyte Ratio; FEV1%: Forced Expiratory Volume in 1 s (percentage); VATS: Video-Assisted Thoracoscopic Surgery; NSCLC: Non-Small Cell Lung Cancer.
